# Acetabular cup positioning in primary routine total hip arthroplasty—a review of current concepts and technologies

**DOI:** 10.1186/s42836-023-00213-3

**Published:** 2023-12-01

**Authors:** Aravind Sai Sathikumar, George Jacob, Appu Benny Thomas, Jacob Varghese, Venugopal Menon

**Affiliations:** 1 Division of Joint Replacement and Sports Medicine, VPS Lakeshore Hospital: Lakeshore Hospital and Research Centre Ltd, Kochi, Kerala 682040 India; 2 Department of Orthopaedics, Bharati Vidyapeeth Deemed University, Pune, Maharashtra 411043 India

**Keywords:** Acetabular cup positioning, Primary THA, Robotic THA, Patient specific instrumentation, Navigation THA, Spinopelvic relation

## Abstract

**Introduction:**

Total hip arthroplasty (THA) has revolutionized the treatment of hip joint arthritis. With the increased popularity and success of the procedure, research has focused on improving implant survival and reducing surgical complications. Optimal component orientation has been a constant focus with various philosophies proposed. Regardless of the philosophy, achieving an accurate acetabular position for each clinical scenario is crucial. In this paper, we review the recent developments in improving the accuracy and ideal positioning of the acetabular cup in routine primary THA.

**Methodology:**

A review of the recent scientific literature for acetabular cup placement in primary THA was performed, with available evidence for safe zones, spinopelvic relationship, preoperative planning, patient-specific instrumentation, navigation THA and robotic THA.

**Conclusion:**

Though the applicability of Lewinnek safe zones has been questioned with an improved understanding of spinopelvic relationships, its role remains in positioning the acetabular cup in a patient with normal spinopelvic alignment and mobility. Evaluation of spinopelvic relationships and accordingly adjusting acetabular anteversion and inclination can significantly reduce the incidence of dislocation in patients with a rigid spine. In using preoperative radiography, the acetabular inclination, anteversion and intraoperative pelvic position should be evaluated. With improving technology and the advent of artificial intelligence, superior and more accurate preoperative planning is possible. Patient-specific instrumentation, navigated and robotic THA have been reported to improve accuracy in acetabular cup positioning as decided preoperatively but any significant clinical advantage over conventional THA is yet to be elucidated.

## Introduction

Total hip arthroplasty(THA) is known as the “Operation of the century” revolutionizing treatment for people suffering from crippling hip arthritis [1]. Accurate orientation and positioning of the acetabular cup in total hip arthroplasty (THA) is crucial for satisfactory outcomes. Erroneous acetabular cup positioning can result in dislocation, accelerated implant wear, osteolysis leading to aseptic loosening of cup, impingement or limb length discrepancy [2, 3]. Anteversion, inclination, height, and offset are important variables during acetabular cup placement [3].

Lewinnek et al. described a safe zone or safe range for the placement of the acetabular component in 1978 [4]. However, recent literature confers poor predictive values for Lewinnek’s safe zone with regard to hip joint instability [5, 6]. It is suggested to consider a “functional safe zone” for acetabular cup placement rather than a “one size fits all” philosophy [5, 6]. Therefore, each case requires preoperative radiological evaluation and planning to determine each individual patient’s functional safe zone [7].

Acetabular cup orientation is significantly influenced by the intraoperative position of patient’s pelvis during THA [8]. Some studies suggest that the use of mechanical guides are superior to freehand techniques for appropriate acetabular cup placement [9]. Further use of preoperative computed topography and 3D printing of custom acetabular jigs decreases the incidence of acetabular cup malposition during THA [10–12].

Navigated THA (N-THA) has been reported to have more accurate positioning of the acetabular component than freehand placement [13, 14]. However, some studies have reported no significant advantage in using navigation for acetabular cup positioning [15, 16]. Robotic THA (R-THA) has been reported to be effective in acetabular cup positioning within the Lewinnek and Callanan safe zones. However, again significant functional difference between robotic vs. conventional THA (C-THA) remains debatable [17–19].

In this paper we aimed to comprehensively review the recent concepts and technological advances for positioning of an acetabular cup in a routine primary THA and their merits, demerits, practicality in clinical application and their functional outcomes.

## Methodology

The PubMed database was searched for recent scientific literature published in last 5 years (2017 to 2022) regarding acetabular cup placement with specific regard to acetabular cup positioning, safe zones for cup placement, spinopelvic relationship in THA, preoperative planning for cup placement, patient-specific instrumentation, navigated and robotic THA. A total of 1,204 articles appeared in the search, out of which 36 eligible articles were considered for critical analysis. We included randomized controlled trials (RCTs), prospective and retrospective case-control or cohort studies which focused on acetabular cup placement in primary routine THA. We excluded narrative reviews, scoping reviews, newsletters as well as other research articles which focused on femoral component positioning, revision THA and acetabular positioning in complex primary THA, such as those for developmental dysplasia of hip, severe protusio acetabuli, previous acetabular surgery, hip infection, severe acetabular bone loss or severe osteoporosis.

### Acetabular cup position and safe zones

Over the last 44 years, the most popular “safe zones” for acetabular cup placement were described by Lewinnek et al. [4]. The Lewinnek safe zone was described based on a series of 300 THAs and has defined the acetabular inclination to be within 40 +/- 10 degrees and the acetabular anteversion cup to be within 15 +/- 10 degrees [4]. More recently, Callanan et al. described safe zones for cup placement, where 1,823 THAs were studied and it was suggested that safe acetabular cup inclination should be within 30-45 degrees, this finding agreeing with the Lewinnek’s safe zone [20]. In 2019, Dorr et al. published an editorial commentary titled Death of Lewinnek “Safe Zone”. He suggested the need for a “Functional Safe Zone” for acetabular cup placement over the traditional Lewinnek safe zone [5]. The concept of a functional safe zone refers to a patient-specific safe zone to avoid instability or impingement which is dependent on various patient-specific factors [5]. However, instability or impingement with regard to the Lewinnek safe zone was described only in patients with abnormal spinopelvic mobility [5, 6, 21]. In cases where spinopelvic mobility cannot be evaluated, for example in an acute femoral neck fracture, where THA is the intended treatment, assessment of spinopelvic mobility with radiographs in the sitting and standing position is not possible. In such situations, the Lewinnek or Callanan safe zones remain an important guide. Therefore, Lewinnek or Callanan safe zones remain applicable for acetabular cup placement in patients where any abnormal spinopelvic mobility has been ruled out preoperatively or where spinopelvic relationships cannot be assessed.

Some surgeons prefer the transverse acetabular ligament (TAL) as a reference to determine acetabular cup anteversion, height and offset [3, 22, 23]. Using the TAL as a reference helps the surgeon note the native anteversion of the acetabulum, which will be variable for each patient and is independent of the patient’s pelvic position [3]. Using the TAL as a landmark is a useful aid for cup positioning, but identification of the TAL is variable and the ligament is difficult to identify in some cases or even absent [3].

### Spinopelvic considerations

**Fig. 1:**
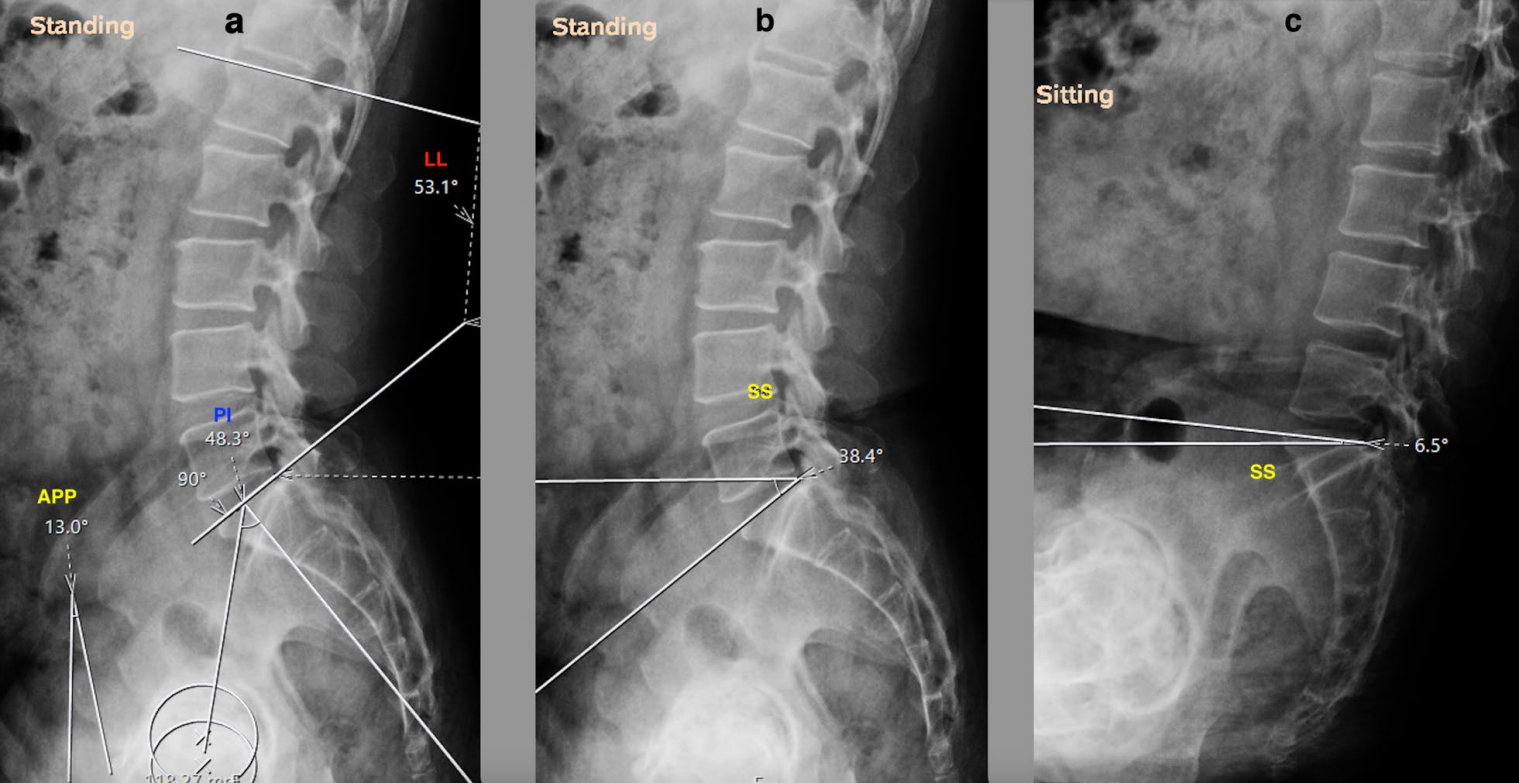
Lumbosacral spine with pelvis lateral view showing normal spine with normal mobility (1A). **a** Pelvic tilt (APP = 13°), PI-LL (48.3°–53.1° = -4.8°). **b**, **c** ^SS > 10°

**Fig. 2:**
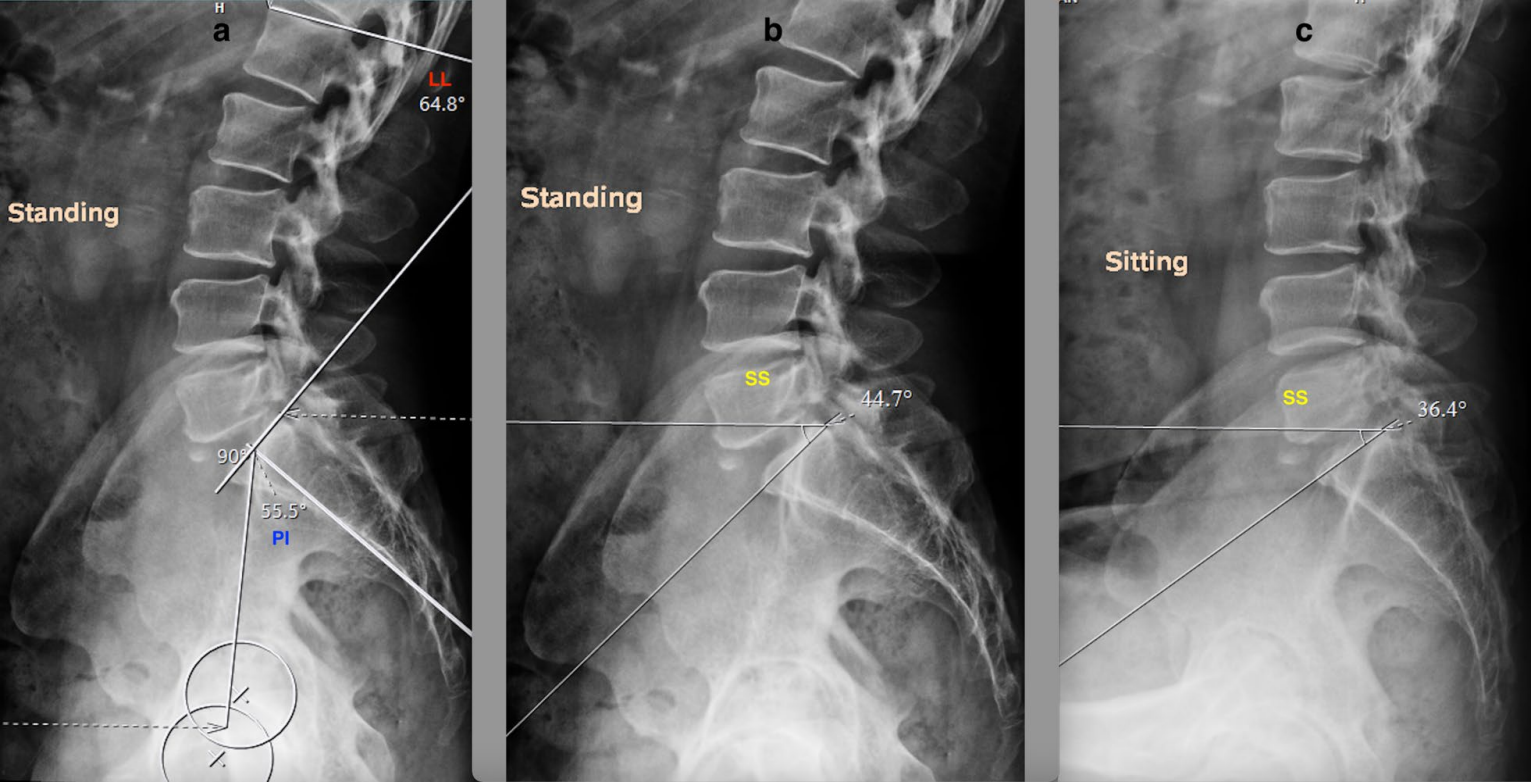
Lumbosacral spine with pelvis lateral view showing a normal spine with reduced mobility (1B). **a** PI–LL (55.5°–64.8° = -9.3°). **b**, **c** ^SS < 10°

**Fig. 3:**
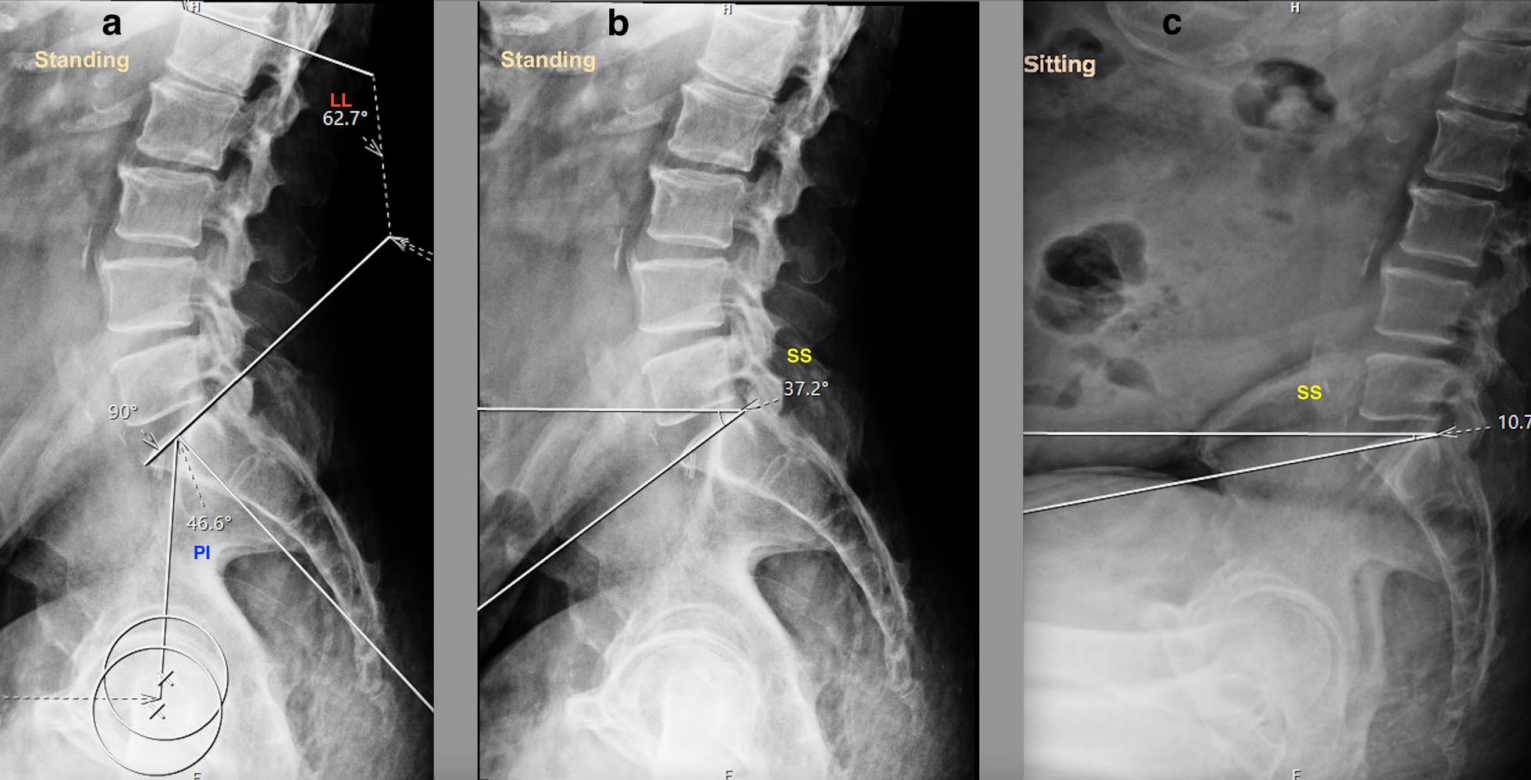
Lumbosacral spine with pelvis lateral view indicating a flatback with normal mobility (2A). **a** PI-LL (46.6°–62.7° = -16°). **b**, **c** ^SS > 10°

**Fig. 4:**
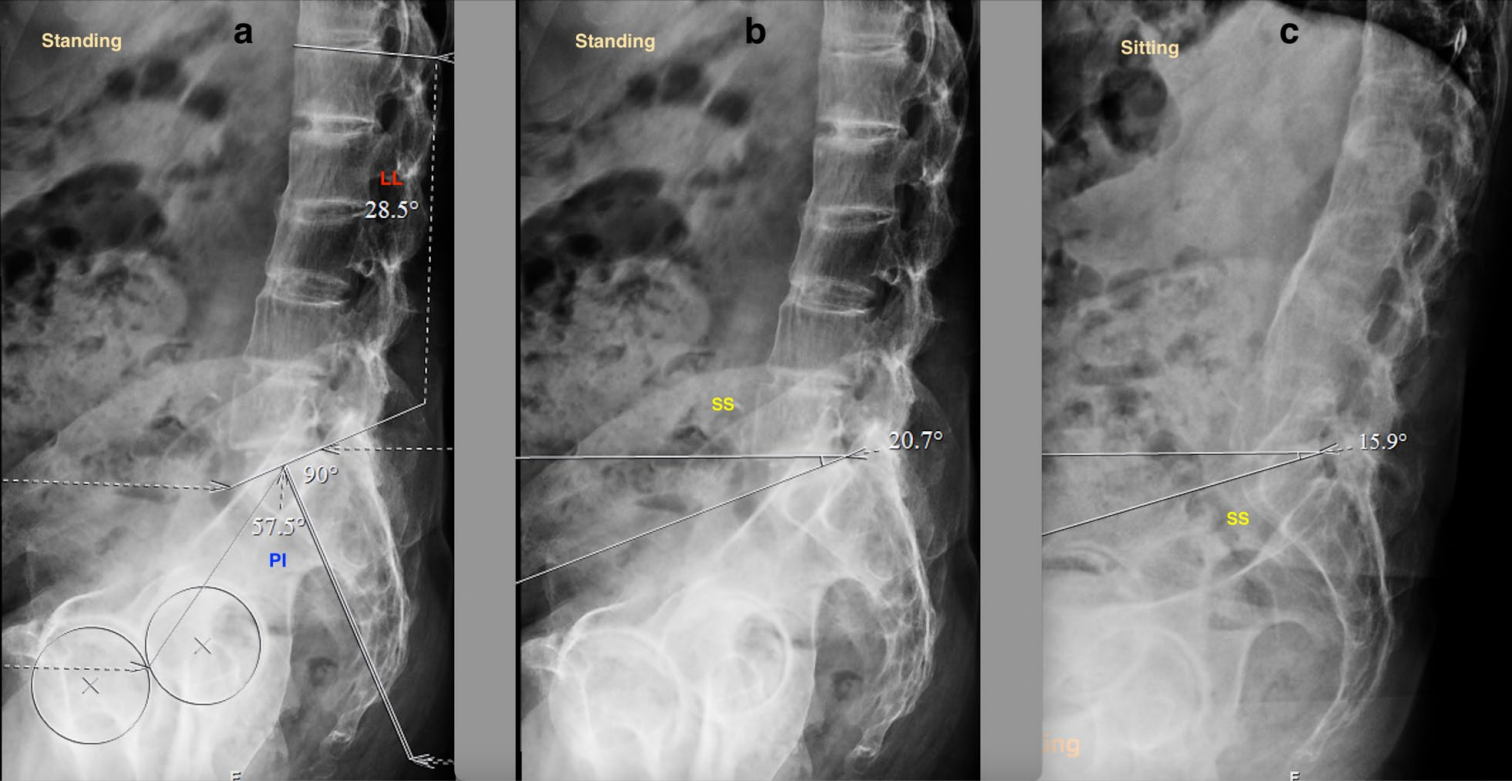
Lumbosacral spine with pelvis lateral view showing a flatback with reduced mobility (2B). **a** PI-LL (57.5°–28.5° = 29°). **b**, **c** ^SS < 10°

Spinopelvic mobility evaluation is important in patients undergoing THA in view of the increased risk of instability and impingement [24, 25]. Two goals are defined to evaluate the spinopelvic relation: (1) to identify the spinal deformity, (2) to identify the spinal stiffness [26]. Spinal deformity is evaluated by measuring the difference between pelvic incidence (PI) and lumbar lordosis (LL) in a standing lateral view of the lumbosacral spine with pelvis and hips (Fig. 1a) [26]. PI is the angle between the line perpendicular to the superior endplate of the S1 vertebra and the line from the S1 vertebra to the center of hips in lateral views (Fig. 1a). LL is the angle between the superior endplates of the S1 vertebra and L1 vertebra (Fig. 1a). If PI-LL (pelvic incidence minus lumbar lordosis) is within -10° to +10°, it is a normal spinopelvic alignment. Whereas if PI-LL is >10°, it is a flatback deformity. Assessment of spinal stiffness is performed preoperatively by determining the change in sacral slope (^SS) from standing to seated lateral radiographs of the lumbosacral spine plus the pelvis (Fig 1b, c), where ^SS < 10° is classified as stiff spine [7, 26–28]. The pelvic tilt, measured as the angle of an anterior pelvic plane (APP) in the lateral spine with pelvic X-ray can also be considered for identifying spinal deformity (Fig 1a) [7]. In the Hip-Spine Classification by Vigdorchik et al., the patients were classified, based on these radiographs, into:

- 1A (Fig. 1)—normal alignment with normal mobility;
- 1B (Fig. 2)—stuck standing (fixed lumbar lordosis that doesn’t change when patient sits, ^SS < 10°);
- 2A (Fig. 3)—flatback with normal mobility (PI-LL > 10, ^SS > 10°);
- 2B (Fig. 4)—stuck sitting (fixed flatback deformity that doesn’t change when patient stands up, PI-LL > 10°, ^SS < 10°) [26, 28].

Based on this, Sharma and Vigdorchik et al., McKnight et al. and Luthringer and Vigdorchik et al. have suggested acetabular cup inclination and anteversions to avoid impingement and instability (Table 1) [7, 27].

**Table 1:** Suggested acetabular inclination and anteversion *as per* various authors based on spinopelvic mobility

**Type**	**Sharma and Vigdorchik et al.**		**McKnight et al.**		**Luthringer and Vigdorchik et al.**
	**Inclination**	**Anteversion**	**Inclination**	**Anteversion**	**Anteversion in standing pelvis AP X-rays**
**1A**	40°	20°–25°	35°–45°	15°–25°	20°–25°
**1B**	45°	25°–30°	45°–50°	20°–25°	30°
**2A**	40°	Anterior pelvic tilt-20°–25°; Posterior pelvic tilt-25° ^a^	40°–45°	20°–25°	25°–30°
**2B**	40°	25° ^a^	35°–40°	15°–20°	30°

^a^but if posterior pelvic tilt >= 13°, then keep anteversion to be less than native anteversion

In recent literature, an increase in anteversion is recommended to avoid impingement or instability in case of abnormal hip-spine mobility or stiffness, especially in stuck sitting patients (2b). This is with respect to the functional pelvic plane evaluated by a standing pelvis AP view [7, 28, 29]. Mcknight et al. recommended a lower range of anteversion for the stuck standing (2B) patients [27]. So, at present, the more ideal guideline to follow is that of Sharma and Vigdorchik et al. where both the functional pelvic plane and pelvic tilt is considered [7]. Most publications have suggested the use of dual mobility cups in patients with stiff spines, especially in the stuck sitting group to mitigate the risk of impingement or dislocation [7, 26–29]. Hence, careful preoperative planning and assessment of spinopelvic relations, even in patients with no complaints or pre-existing diagnosis with respect to spine are necessary to decide on anteversion and inclination of acetabular cup placement [7, 26–29]. Gu et al. (2021), in their study to evaluate possible impingement after THA, used a combination of mathematical calculations and automated computational simulations preoperatively. They were able to report the various possible cup orientations which could cause impingement. Navigation THA fails to consider spinopelvic mobility, thereby permitting a risk of impingement and instability in patients with abnormal spinopelvic mobility [30]. Current robotic THA systems that predict cup position, take into consideration the sacral slope and pelvic tilt (calculated from plain radiographs). A preoperative planning software that incorporates complete sagittal, coronal, and transverse axes into consideration to determine a patient-specific functional safe zone could perform a truly kinematic THA.

### Preoperative planning

**Fig. 5:**
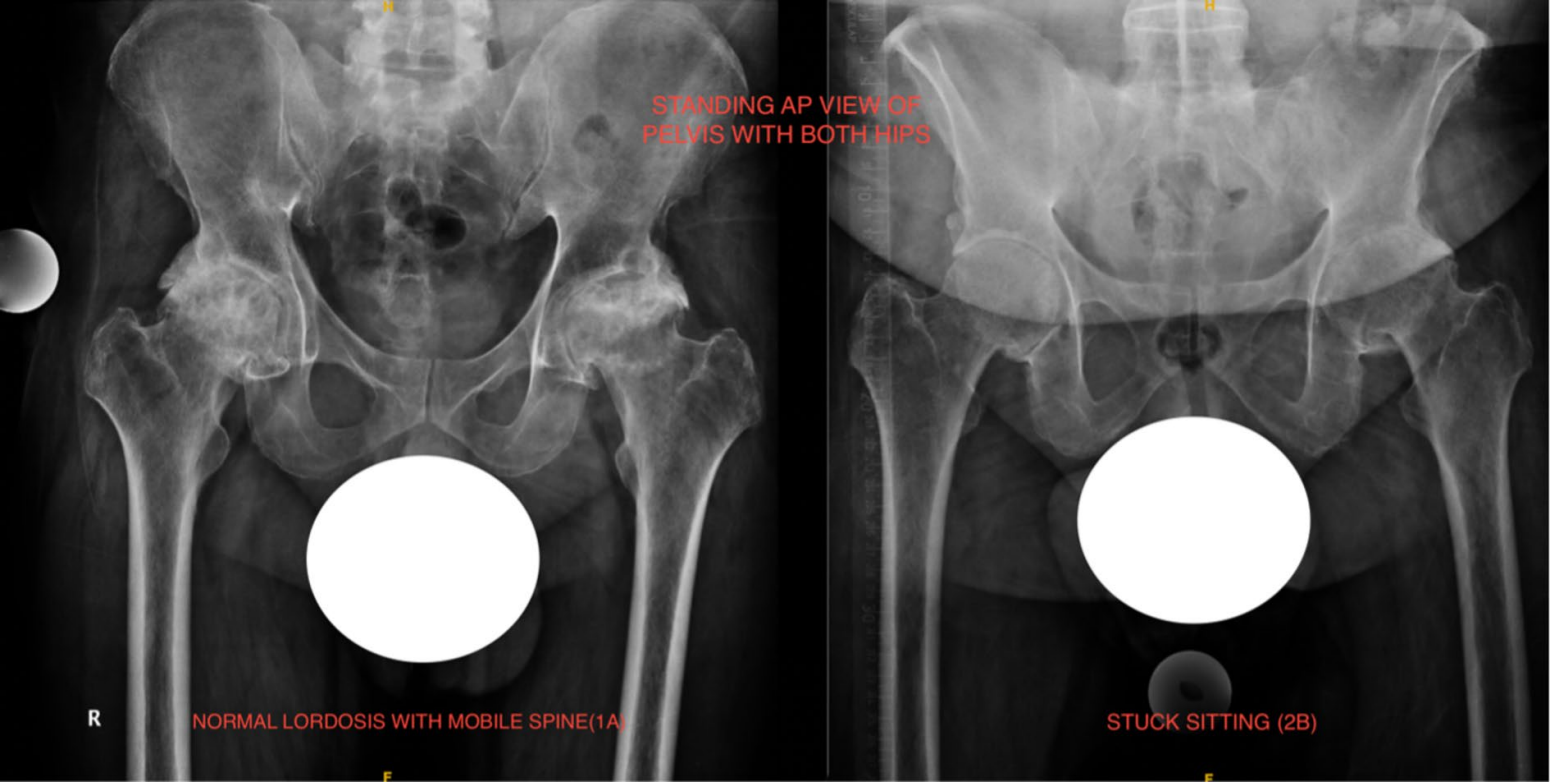
Standing AP view of pelvis with both hips. (Right)—A patient with normal lordosis and mobile spine. (Left)—A patient with flat back deformity and immobile spine

**Fig. 6:**
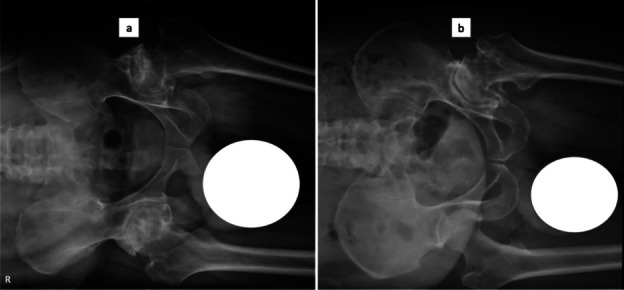
Pelvis with both hips AP view with the patient lying in lateral position to evaluate the position of the pelvis at the time of surgery—**a** No coronal plane deformity, iliac crest at the same level indicating pelvis is perpendicular to the horizontal plane; **b** Hip abduction deformity showing iliac crest at different levels hence pelvis is tilted in lateral position

Preoperative planning for a conventional THA, imageless navigation or imageless robotic system should include appropriate radiography to determine and execute accurate acetabular cup positioning. Radiographs required would be standing anteroposterior (AP) views of pelvis with both hips (Fig. 5), a pelvis AP view with the patient in lateral decubitus if surgery is done in this position (Fig. 6), sitting, and standing lumbosacral spine and pelvis lateral views (Figs. 1, 2, 3, 4). AP views should include a marker for magnification reference allowing for accurate templating on a printed film. Software that aids in preoperative templating should utilize a standing pelvis AP view as this gives a functional pelvic plane [7, 28]. AP view of pelvis with the patient lying in lateral recumbency simulates the intraoperative position of the pelvis in the lateral decubitus. Beverland et al. described the operative inclination for a patient in the lateral position and reported difficulty in achieving a squared pelvis in the lateral position [3]. Coronal plane deformities such as scoliosis, abduction or adduction deformities of hip also affect pelvic position when the patient lies in the lateral position, and this can lead to erroneous cup inclination, if it is not identified preoperatively. Sitting and standing lumbosacral spine radiographs including the pelvis help assess the spinopelvic relations as discussed earlier. The stuck sitting group (2b) should be approached and planned with extra caution. EOS^®^ imaging systems provide whole body biplanar functional imaging with low radiation and has been reported to be more accurate than conventional radiographs for preoperative templating [31].

A few navigation and robotic systems use preoperative computed comography (CT) for predicting the acetabular cup position, but the above-mentioned plain radiographs still have their importance as the patients’ functional pelvic plane and intraoperative pelvic position can affect the cup inclination and anteversion.

### Role of patient-specific instrumentation

PSI in THA involves using acetabular guidance systems aimed to achieve accurate cup size, inclination and anteversion using a preoperative plan [11]. The plan is developed using preoperative MRI or CT scans, both of which seem to be equally effective. However, MRI causes no radiation exposure [11]. Most reports state an increased operative time and cost when using PSI in THA when compared to C-THA and any clinical advantage over C-THA is unclear [10, 11, 32]. Published studies do suggest that PSI is useful in THA to improve accuracy in acetabular cup position as preoperatively planned as compared to C-THA [12, 32, 33]. PSI may therefore have a valuable role in patients with complex and abnormal spinopelvic relationships, where a functional safe zone of acetabular cup placement is important [12, 34].

Five studies were evaluated to determine the efficacy of PSI-THA (Table 2). These included 2 RCTs, 1 case-control study and 2 cohort studies. In the RCTs and the case-control study, the authors have compared the postoperative acetabular cup anteversion and inclination using PSI versus cup position in C-THA [10, 12, 33]. In the other studies, they compared the efficacy of PSI in achieving the preoperative plan [32, 34]. Two studies reported functional outcomes and noted no significant difference between PSI-THA and C-THA [12, 33]. In the study by Inoue D et al., the PSI was designed based on MRI, whereas in the remaining 4 studies, PSI was designed based on CT. Though all the studies reported that PSI-THA was a safe technique for acetabular component positioning without major outliers, they did not conclude that PSI is superior to C-THA [10, 12, 32–34]. Chen X et al. reported that PSI-THA achieved acetabular inclination and anteversion more accurately (<5° of preoperative target) than C-THA but failed to demonstrate any functional superiority [33].

**Table 2:** Studies which evaluated the role of PSI-THA

**Author**	**Study Type / Number of Patients**	**Study Group 1**	**Study Group 2**	**Outcome**	**Results**
Inoue D. et al., [10]	Prospective cohort study / 14 hips	Preoperative templated CT	Postoperative CT	Postoperative CT of patients who underwent THA with MRI based PSI for acetabulum was compared to their preoperative templating.	Inclination angle in all cases were within +/-10° of preoperative templated angle but with respect to anteversion, 3 cases were outliers. After an initial learning curve all cases were within the desired target range.
Mishra A. et al., [10]	Prospective RCT / 36 hips	PSI- THA	C-THA	Postoperative X-rays were evaluated for cup anteversion and inclination.	No statistically significant difference in acetabular cup inclination and anteversion angles between the 2 groups was found.
Ferretti A. et al., [34]	Prospective cohort study / 36 hips	Preoperative templated CT	Postoperative CT	Postoperative CT of patients who underwent THA with CT based PSI with laser for acetabulum was compared to their preoperative templating.	No statistically significant difference in inclination and anteversion of acetabular cup in postoperative CT was found as compared to preoperative planning.
Thomas C. et al., [12]	Prospective RCT / 51 hips	PSI-THA	C-THA	Postoperative CT was evaluated for differences in cup anteversion and inclination between the 2 groups. Functional outcome between the 2 groups up to 12 months were evaluated.	No significant difference in mean acetabular anteversion or inclination was found in both the groups. Though a greater number of outliers (>10° than preoperatively targeted) were present in C-THA group, this was not statistically significant. No difference in functional outcome was found at follow-up.
Chen X. et al. [33]	Prospective case-control study / 60 hips	PSI-THA	C-THA	Postoperative X-rays were evaluated for cup anteversion and inclination. Functional outcomes evaluated at 4 and 12 weeks. Femoral parameters were also noted.	Statistically significant number of patients achieved accurate (<5° as preoperatively targeted) inclination and anteversion with PSI-THA as compared to C-THA. But no significant difference in functional outcomes was noted.

### Role of navigation THA

Computer-assisted surgery (CAS) or navigation total hip arthroplasty (N-THA) was first introduced 3 decades ago and the technology has been constantly evolving [35]. N-THA is a dependable tool for acetabular cup placement, but has drawbacks of additional surgical time and cost of procedure [35]. N-THA systems were introduced as a CT-based guiding system and more recently imageless navigation systems have become available [35, 36]. Recent literature suggests significantly better safe zone positioning of the acetabular component with use of N-THA [13, 14, 16, 37–41]. Among these studies, most authors reported a more accurate placement of the acetabular component with respect to the anteversion [39–42]. Studies comparing functional outcomes or the complications between C-THA and N-THA have found comparable results between the 2 groups [14, 38, 41, 42]. Tanino H et al. (2020) noted that there was a significant increase in operative time with the use of N-THA [37]. An increase in operative time might be attributed to the pin placement, landmark registration time and time taken for the system to process the information. In 2012, Sugano et al. reported a 100% survival rate of ceramic-on-ceramic THAs done with N-THA against a 95.6% survival rate with the use of C-THA at 13-year follow-up [43]. Further long-term studies with large sample sizes are necessary to assess the functional outcomes and complications of N-THA as compared to C-THA to prove a significant advantage of one over the other.

We analyzed 9 studies that compared N-THA and C-THA (Table 3). Four were case-control studies, 4 were randomized controlled trials (RCT) and 1 was a comparative study. All these studies were aimed at identifying the accuracy of acetabular cup placement using N-THA versus C-THA. Four studies included functional outcomes or complications also in their outcome results [14, 38, 41, 42]. Only one study concluded that there was no significant difference between component placement using N-THA or C-THA, but this study had a relatively smaller sample size [16].

**Table 3:** Studies comparing N-THA vs. C-THA

**Author**	**Study Type / Number of Patients**	**Study Group 1**	**Study Group 2**	**Outcome / Follow-up**	**Results**
Jacob I et al., [13]	Case-Control study / 102 hips	N-THA	C-THA	Radiographs were evaluated for cup placement within safe zones (Lewinnek).	Cup inclination and anteversion were more consistent in N-THA group.
Naito Y et al., [14]	Case-Control study / 184 hips	N-THA	C-THA	CT scans were evaluated for cup placement within safe zones(Lewinnek). Complications were followed up.	Significantly higher percentage of acetabular cups were within safe zones using N-THA. But no difference was found in incidence of hip dislocation.
Tanino H et al., [37]	Prospective RCT / 110 hips	N-THA	C-THA	Radiographs were evaluated for cup placement within safe zones (Lewinnek).	Significantly higher percentage of acetabular cups were within safe zones using N-THA. But there was statistically significant increase in average operative time with N-THA.
Nishihara S et al., [38]	Case-Control study / 144 hips	N-THA	C-THA	CT scans were evaluated for cup placement within safe zones. Dislocations within 6 months of postoperative period were noted.	N-THA had less number of cases outside the targeted zone. One case in C-THA group developed posterior dislocation.
Takada R et al., [39]	Comparative study / 30 hips	Navigation assessment	Manual Goniometer Assessment	Evaluation using CT scan at 3 months for the absolute error in inclination and anteversion measured using navigation or goniometer intraoperatively.	Absolute estimate error of cup anteversion in navigation measurement was significantly lower than in goniometer measurement.
Okamoto M et al., [42]	Case-Control study / 221 hips	N-THA	Alignment Guide THA	CT scans were evaluated for cup placement within safe zones(Lewinnek). Functional outcome was evaluated.	Anteversion errors were less in N-THA group. No significant differences in functional outcomes was found.
Mihalic R et al., [40]	Prospective RCT / 84 hips	N-THA	C-THA	CT scans were evaluated for cup placement within safe zones(Lewinnek).	Less number of outliers from safe zone in N-THA group and significantly higher accuracy for anteversion was noted using N-THA.
Ogawa H et al., [16]	Prospective RCT / 41 hips	N-THA	C-THA	Radiographs and CT scans were evaluated for cup placement within safe zones (Lewinnek).	No significant differences were found in component placement using either methods.
Lass R et al., [41]	Prospective RCT / 110 hips	N-THA	C-THA	Radiographs were evaluated for cup placement at 6 weeks followup. Functional outcomes were evaluated for up to 2 years.	Significant difference in mean anteversion but no difference was noted in mean inclination. No difference in functional outcomes was found.

### Role of robotic THA

Over the last decade, robotic THA (R-THA) has become popular with the expectation of more accurate component positioning and therefore improved functional outcomes and implant survivorship [44]. Nodzo et al. (2018) reported that R-THA is a reliable system with respect to intraoperative acetabular component position, as the intraoperatively aimed anteversion and inclination correlated with the postoperative component position [45]. Recent literature comparing R-THA with conventional THA has reported more accurate acetabular cup placement within the safe zone (Lewinnek or Callanan safe zones) using R-THA [17–19, 46–48]. Shaw J. H. et al. (2021) reported a reduced surgical duration, hospital stay and dislocation rate with robotic THA [46]. Kayani et al. (2021) studied the average learning curve of R-THA’s. They reported that, after 12 R-THA’s the operative time, and surgical team confidence was comparable to that of a conventional THA [49]. Another group reported (2021) an overall decrease in length of hospital stay and cost incurred at 1 year with R-THA, but found no differences in surgical complications when compared to conventional THA [50]. The reason for reduced surgical time could be due to more intensive preoperative planning allowing surgeons to have a better idea of the acetabular geometry and implant requirements [51]. From an intraoperative perspective, surgical time could be reduced in view of a single reaming using the robotic arm, rather than serial reaming, as in conventional THA. Despite these advantages, at present, there is a lack of long-term functional outcomes to declare that R-THA is superior to navigation or conventional THA. Considering the additional cost for procuring the Robot, R-THA may be best used in complex cases at a referral centre.

We analysed 8 studies which compared R-THA and C-THA (Table 4), including 6 case-control studies, 1 cohort study and 1 RCT. All these studies compared the accuracy of component positioning using R-THA vs. C-THA. Four studies also compared the complications or functional outcomes in the two groups [17–19, 46]. Domb BG et al., conducted a case-control study with propensity score match between the 2 study groups and reported superior functional outcomes for R-THA patients over C-THA at a midterm follow-up.

**Table 4:** Studies comparing R-THA vs. C-THA

**Author**	**Study Type / Number of Patients**	**Study Group 1**	**Study Group 2**	**Outcomes**	**Results**
Shaw J.H. et al., [46]	Cohort Study/2247 hips	R-THA	C-THA	Number of dislocations and revision surgery for instability was noted. Minimum follow-up of 6 months duration. A representative sample of (368 hips) X-rays were assessed for cup anteversion and inclination.	Robotic group had reduced surgical duration, hospital stay and dislocation rates. Robotic group had greater anteversion, but less inclination than conventional THA.
Stewart N.J. et al., [47]	Case-control study/200 hips	R-THA	Fluoroscopy assisted THA	Evaluation for cup placement within safe zones (Lewinnek and Callanan)	Greater percentage of cases from robotic group fell into safe zones as compared to fluoroscopy assisted groups.
Li Y et al., [52]	Case-control study/246 hips	R-THA	C-THA	Evaluated for cup placement within safe zones(Lewinnek and Callanan).	No significant differences found in cup position within safe zone
Foissey C et al., [17]	Case-control study/150 hips	R-THA	C-THA	Acetabular cup inclination, anteversion, offsets were measured. Harris hip score(HHS) and complications were assessed at 1 year.	Centre of rotation was more accurately restored with R-THA. No difference in functional outcome or complications at 1 year follow-up.
Zhang S et al., [18]	Case-control study/116 hips	R-THA	C-THA	Acetabular cup inclination, anteversion, offsets were measured at 3, 6 and 12 months along with functional outcomes in obese patients.	Greater percentage of cases using R-THA achieved targeted angles as compared to C-THA. Functional outcomes were comparable in both groups.
Domb B.G. et al., [19]	Case-control study/132 hips	R-THA	C-THA	Acetabular cup inclination, anteversion, offsets were measured. Functional outcomes were evaluated and had a minimum of 5 year follow-up.	Greater percentage of cases using R-THA were in safe zones as compared to C-THA group. Global offset and functional outcomes were also better with R-THA
Wang W. et al., [48]	Prospective RCT/72 hips	R-THA	C-THA	Acetabular cup inclination, anteversion, offsets were measured.	Cup anteversion within safe zone was found to be better with R-THA, but no difference was noted in cup inclination.

## Discussion

Lewinnek and Callanan safe zones have been regarded as the standard of practice at several centres for many years and have recently been contested on their applicability. This has arisen due to better understanding of spinopelvic relationships and motion. Surgeons have reported increased frequency of hip impingement and dislocation in patients with fused lumbar spines or patients with abnormal spinopelvic mobility [53–55]. We understand that Lewinnek and Callanan safe zones are still applicable when implanting an acetabular component where the patient has normal spinopelvic mobility. Additionally, the TAL is also a useful intraoperative reference point for cup placement and can help guide cup anteversion matched to the patient’s native anatomy [3].

Patients with rigid spines are at a higher risk of dislocation when compared to those with normal spinal biomechanics. Acetabular cup positioning must therefore be carefully planned, especially, in the stuck sitting group (2b). Pelvic tilt, PI-LL and change in sacral slope must be assessed preoperatively and acetabular cup anteversion should be judiciously planned to avoid impingement and an unstable hip. Preoperative radiological evaluation is very important to the understanding of the spinopelvic relationship and functional pelvic plane. It is also worth getting radiographs in the anticipated intraoperative position of the pelvis.

Preoperative planning of cup anteversion and inclination are important, but the execution of these predetermined angles with accuracy poses another challenge. PSI-THA, N-THA and R-THA are methods used to improve intraoperative accuracy of acetabular cup placement. PSI-THA is a reliable method to reconstruct the preoperatively planned acetabular cup position during surgery and hence could be beneficial in cases with complex or abnormal spinopelvic relationship [12, 34]. N-THA is reported to be superior, in achieving more accurate acetabular cup placement, to C-THA but increase the operative time [13, 14, 37, 38]. R-THA improves acetabular cup placement and has a shorter duration of surgery than C-THA [17–19, 46, 48, 51]. Singh et al. compared the early patient-reported outcome measures (PROMs) of C-THA, N-THA as well as R-THA and noted better patient-reported outcomes with C-THA, but concluded that there were no significant differences between the 3 groups [56]. Differences in functional outcomes between PSI-THA, R-THA, N-THA and C-THA have yet to be reported in long-term studies. R-THA software continues to evolve and now some researchers also take spinopelvic mobility into consideration while deciding the final cup anteversion and inclination. There have been reports on conversion of R-THA to C-THA due to technical difficulties during surgery [57, 58]. Hence, we should also understand that before a surgeon starts using N-THA or R-THA, they should be well familiar with a C-THA. Technology should be an aid but not a substitute for the surgical judgment. Therefore, a clear understanding of the acetabular cup placement taking all the factors into consideration and accurate intraoperative cup placement is important for achieving a stable hip post-operatively.

Unstable THA is classified into 6 types: (I) acetabular malposition, (II) femoral component malposition, (III) abductor deficiency, (IV) impingement, (V) late poly wear, (VI) unclear etiology [59]. The above-mentioned components are not always isolated, and a combination of these factors leads to an unstable hip. Femoral anteversion and combined anteversion are very important factors to consider during THA but are beyond the scope of this paper.

## Conclusion

THA has revolutionized the management of hip joint arthritis. Research has been focusing on further lowering complication rates and improving implant survivorship. To reduce complications, preoperative planning and spinopelvic relations must be evaluated in all patients undergoing THA with special attention paid to the stuck sitting (2b) group. PSI-THA, N-THA and C-THA have been the technological advances that improve acetabular cup positioning. However, the long-term functional superiority of these to C-THA warrants further study. R-THA might be used for complex THAs but routine primary THA cases can be managed with C-THA satisfactorily. In centers without facility for N-THA or R-THA, PSI-THA could be considered a viable option for the management of cases with complex spinopelvic relationships. As per registry data, about 3/4 of cases that undergo THA have a 15-20 year survivorship and >50% of the THAs achieve 25-year survivorship [60]. Surgeons should plan preoperatively and determine accurate component positioning intraoperatively to improve surgical outcomes and implant survivorship.

## Data Availability

Data are available from the corresponding author on reasonable request.

## Abbreviations

AP: Antero-Posterior
APP: Anterior Pelvic Plane
CAS: Computer Assisted Surgery
CT: Computed Tomography
C-THA: Conventional Total Hip Arthroplasty
MRI: Magnetic Resonance Imaging
N-THA: Navigation Total Hip Arthroplasty
PI-LL: Pelvic Incidence minus Lumbar Lordosis
PROM: Patient Reported Outcome Measures
PSI: Patient Specific Instrumentation
RCT: Randomised Controlled Trials
R-THA: Robotic Total Hip Arthroplasty
^SS: Change in Sacral Slope
TAL: Transverse Acetabular Ligament
THA: Total Hip Arthroplasty
